# Transcranial Magnetic Stimulation and Neocortical Neurons: The Micro-Macro Connection

**DOI:** 10.3389/fnins.2022.866245

**Published:** 2022-04-12

**Authors:** Dongting Tian, Shin-Ichi Izumi

**Affiliations:** ^1^Department of Physical Medicine and Rehabilitation, Tohoku University Graduates School of Medicine, Sendai, Japan; ^2^Graduate School of Biomedical Engineering, Tohoku University, Sendai, Japan

**Keywords:** motor cortex, neurocytology, neurophysiology, glutamate, GABA, transcranial magnetic stimulation (TMS), neurons

## Abstract

Understanding the operation of cortical circuits is an important and necessary task in both neuroscience and neurorehabilitation. The functioning of the neocortex results from integrative neuronal activity, which can be probed non-invasively by transcranial magnetic stimulation (TMS). Despite a clear indication of the direct involvement of cortical neurons in TMS, no explicit connection model has been made between the microscopic neuronal landscape and the macroscopic TMS outcome. Here we have performed an integrative review of multidisciplinary evidence regarding motor cortex neurocytology and TMS-related neurophysiology with the aim of elucidating the micro–macro connections underlying TMS. Neurocytological evidence from animal and human studies has been reviewed to describe the landscape of the cortical neurons covering the taxonomy, morphology, circuit wiring, and excitatory–inhibitory balance. Evidence from TMS studies in healthy humans is discussed, with emphasis on the TMS pulse and paradigm selectivity that reflect the underlying neural circuitry constitution. As a result, we propose a preliminary neuronal model of the human motor cortex and then link the TMS mechanisms with the neuronal model by stimulus intensity, direction of induced current, and paired-pulse timing. As TMS bears great developmental potential for both a probe and modulator of neural network activity and neurotransmission, the connection model will act as a foundation for future combined studies of neurocytology and neurophysiology, as well as the technical advances and application of TMS.

## Introduction

Neurons in the developed neocortex are highly diversified in their morphology, distribution, connection, and physiological function in the central nervous system. Approximately 70–85% of the neocortical neurons are excitatory, projecting across cortical and subcortical areas and promoting cortical signal propagation (DeFelipe et al., 2002). The remaining 15–20% are highly heterogeneous cortical inhibitory interneurons (INs) that project onto local excitatory neurons (Wonders and Anderson, 2006; Benarroch, 2013). Generally, the neocortical neural network consists of the excitatory system and the inhibitory system; the two major neurotransmitters are glutamate and GABA (Benarroch, 2013). The structure of the neocortex has been evidenced by numerous cytoarchitectonic and myeloarchitectonic research on human and other mammals; a “six-layered laminar structure” with large neuron taxonomy is reported (Brodmann, 1909; Amunts and Zilles, 2015; Palomero-Gallagher and Zilles, 2019). The resolution of the neuron landscape was further increased by neurochemical tracing of specific protein and neuropeptide expression, from which the neural response to intracellular and extracellular environmental alteration was also unveiled (Banasr et al., 2017).

The excitatory–inhibitory balance in the human primary motor cortex (M1) can also be assessed and modulated with transcranial magnetic stimulation (TMS) (Barker et al., 1985). Although the macroscopic TMS response represents the collective activity of massive neurons and neurocircuitries (Sanchez-Vives et al., 2017), the investigation of specific circuits can be achieved by varying the TMS pulse parameter and paradigm. For example, the intracortical and interhemispheric excitatory (facilitatory) and inhibitory phenomena mediated by glutamatergic and GABAergic circuitries have been widely investigated using the paired-pulse TMS paradigm (Ferbert et al., 1992; Kujirai et al., 1993). Meanwhile, epidural recordings of TMS-evoked corticospinal descending volleys, as the most direct demonstration of the TMS-evoked aggregative cortical activities, have also provided valuable evidence indicating the properties of the cortical neural circuits involved in TMS (Di Lazzaro et al., 1999; Kallioniemi et al., 2018; Higashihara et al., 2020).

Experimental evidence from TMS studies has indicated a close relationship between TMS outcomes and the cortical neural network (including the neurotransmitters glutamate and GABA) (Premoli et al., 2014; Du et al., 2018). However, as molecular and neurobiological studies mainly focus on the cytoarchitecture (Boyle et al., 2017; Llorca et al., 2019) and cytochemistry (Adotevi and Leitch, 2017) of the neocortical neurons, it remains to be elucidated how the macroscopic electrophysiological response connects and interacts with the microscopic neural circuitry. Specifically, what subsets of cortical network participate in the TMS-evoked response and contribute to the electrophysiological phenomena? Where are the TMS-related circuits and neurons located in the neocortex and how do they interact? Through what neuron population and connection does the TMS-related neural network function? Ultimately, is there a perspective able to connect the macroscopic and microscopic evidence between M1-TMS and M1 cortical neurons? To answer these key problems, an explicit model connecting the evidence from brain stimulation and cortical neural architecture is required. Indeed, establishing a micro–macro connection model between cortical neural circuits and TMS, despite the considerable difficulties so far encountered, is not only essential for exploring the TMS mechanisms in the resolution of neurons, but is also a key issue to be addressed in deciphering the topology and wiring of the normal human cortical network. As TMS is a promising neuromodulation technology applied worldwide, elucidating its underlying mechanisms is also of vital importance for optimizing the outcomes of its use in neurorehabilitation.

In the present integrative review, we focus particularly on the micro–macro connection between cortical neurons in the human motor network (with an emphasis on M1) and TMS to first address the aforementioned questions. By reviewing a large body of literature in the disciplines of neocortical neurocytology and brain stimulation, we seek to establish a model regarding the underlying neural mechanisms of TMS, which is able to bridge the TMS-related electrophysiological and cellular mechanisms while fitting the major experimental evidence. In particular, we reviewed a wider range of neurocytologic literature that seems to have less relationship with TMS for two reasons. First, concerning the shared common research target of the two disciplines—the cortical neurons, we believe that with the knowledge of the microstructure of the cortex, a great leap forward in neuroscience can be expected if we can connect the micro-macro evidence regarding the cortical neurons. Second, by reviewing the developmental and taxonomical evidence, we intend to generate a wider fundament for the micro-macro connection at the preliminary stage, as there may be further involvement of the neuron types other than those included in our preliminary model that has not been identified.

## Methods

### Search Strategy

Based on the integrative review strategy, the author DT searched the electronic databases of PubMed, PubMed Central (PMC), Science Direct, Scopus, Google Scholar, and Web of Science in December 2021. The search terms included the combinations of variants of: “GABA,” “glutamate,” “cortical neurons,” “cortical layers,” “transcranial magnetic stimulation,” “TMS,” “paired-pulse TMS,” “motor cortex,” “I-wave,” “interneurons,” and “cortical neuron hierarchy.” The returned articles were then screened according to the criteria for inclusion and exclusion, duplicates or articles that disqualify the inclusion criteria were excluded.

### Inclusion and Exclusion Criteria

The general inclusion criteria required: (1) peer-reviewed publications (or preprints) with full-text available; (2) publications in English and Japanese; (3) published between January 1, 1993 and November 30, 2021. Criteria for inclusion of neurocytologic articles included (1) human or animal studies with clear ethical statements; (2) study on healthy human or animals (non-pathological studies); and (3) study on the cortical neurons and interneurons. The inclusion criteria of TMS studies required (1) study in healthy adults; (2) TMS of the motor cortex (M1 hand area and the motor-related areas); (3) study with no involvement of neuroplasticity (which can possibly affect the original properties of TMS protocols); and (4) original experimental research (not systematic reviews, dissertations or letters to the editor).

## Cortical Neurons

### Development, Taxonomy, and Morphology

Evidence from mammalian studies has identified that cortical neurons are originally generated at subcortical regions. For excitatory pyramidal neurons (PNs), the developmental origin is known as the radial glia progenitor cells in cortical proliferative pool, located in the proliferative zones (ventricular and subventricular zone, VZ, and SVZ) in primates (Charvet et al., 2017). The newborn neurons migrate under the guidance of the radial fiber through four stages of mitosis and morphological changes and finally arrive at the target neocortical plates (Kriegstein, 2005). For inhibitory INs, a generally accepted origin is the ganglionic eminence (GE, consisting of lateral, medial, and caudal parts: LGE, MGE, and CGE, respectively) in VZ and SVZ. Neuroblasts generated in the GE migrate to the different layers of the neocortex until postnatal stages (Scarabello et al., 2021). In the prenatal and postnatal human brain, neuroblasts migrate from the GE to the neocortex following two main migrating streams, known as the rostral migratory stream (RMS) and the medial migratory stream (MMS) (Sanai et al., 2011; van Strien et al., 2011), and finally differentiate into INs (Faux et al., 2012). The MMS then diverges from the main stream of RMS (heading toward the olfactory bulb) at the anterior horn of the lateral ventricle and heads toward the ventromedial prefrontal cortex, comprising the inhibitory INs in the neocortex (Kubo and Deguchi, 2020).

**FIGURE 1:**
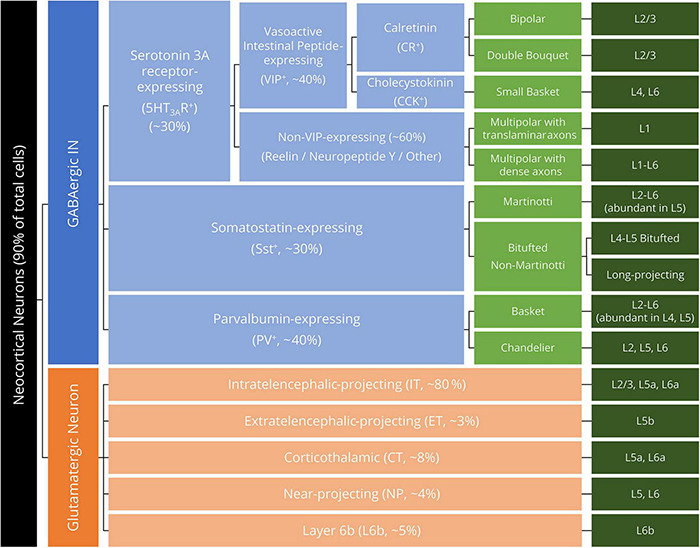
Hierarchical neuron taxonomy of the human motor cortex. All neurons are classified by the neurotransmitter glutamate and GABA as glutamatergic (excitatory) neurons (orange boxes) and GABAergic interneurons (INs, blue boxes). For the morphologically diversified INs, the morphological feature of the IN is specified in light green boxes. Dark green boxes denote the laminar distribution (layer, L) of the listed neuron subclasses.

Regarding the taxonomy of motor cortex neurons in developed rodents and primates, a transcriptome-based taxonomy was recently proposed (Callaway et al., 2021). This hierarchy has emerged from numerous visualization and tracing methodologies for neocortical neurons, including immunocytochemistry (using whole-tissue sections) (Gu et al., 2017), immunohistochemistry (using isolated cells) (Ding et al., 2021), electron (fluorescence) microscopy (Tiveron et al., 2016; Glausier et al., 2021), and single-nucleus RNA sequencing (Krienen et al., 2020). Neocortical neurons are generally categorized by the main neurotransmitter: glutamate for the excitatory PNs and GABA for the inhibitory INs. GABAergic INs are further divided by the expression of specific proteins or neuropeptides, whereas glutamatergic neurons are classified by their laminal distribution and projection patterns (Tremblay et al., 2016; Callaway et al., 2021; Langseth et al., 2021; Figure 1). While the laminar layout and neuron taxonomy are similar across neocortical areas (Douglas and Martin, 2004; Kast and Levitt, 2019), the human primary motor cortex (Brodmann area 4), in particular, distinguishes itself from other cortical areas by the unique presence of giant PNs in layer 5b (L5b, Betz cells), in which the descending axons constitute the core of the corticospinal tract (CST) (Betz, 1874). As further detailed discussions of the development, genetic properties, and pathological alterations of neocortical neurons are beyond the scope of the present review, interested readers are redirected to the comprehensive reviews in these fields (Hu et al., 2017; Tasic et al., 2018; Bakken et al., 2021; Callaway et al., 2021; Matho et al., 2021).

### Neurotransmitter: Glutamate

#### Intratelencephalic Projecting Neurons

Located mainly in L2/3, L5a, and L6a, the IT neurons constitute approximately 80% of the PN population (Bakken et al., 2021; Zhang et al., 2021). In the human motor cortex, the relative proportion of IT neurons is significantly larger than that in mice and marmosets (Bakken et al., 2021), suggesting the essential contribution of IT neurons in the control of human movement. IT neurons can be divided according to the projection area into corticofugal projection neurons (projecting to subcortical structures in both hemispheres), associative projection neurons (projecting to other cortical areas in the ipsilateral hemisphere), and commissural projection neurons (projecting to homologous cortical area in the contralateral hemisphere) (Fame et al., 2011; Greig et al., 2013; Sohur et al., 2014). The L2/3 cortical IT neurons in the motor cortex are mostly commissural and associative projection neurons, which interconnect by synapses, receive input from other cortical areas, and project to the bilateral cortex and striatum or target the L5 extratelencephalic projecting (ET) and IT neurons as a pivot in the sensorimotor integration (Mao et al., 2011; Hooks et al., 2013; Lin et al., 2018). IT neurons in L5a and L6a are mostly corticofugal projecting, with different developmental and functional properties from L2/3 IT neurons (Greig et al., 2013). In the human temporal cortex, the L5 IT neurons display tuftless dendritic morphology and nearby projection property (Kalmbach et al., 2021), whereas the projection of L6 IT neurons in the human motor cortex remains largely unknown. Together, L2–6 IT neurons send their projections within the telencephalon, targeting the bilateral neocortex, striatum, and subcortical structures and, in particular, the contralateral neocortex (Brown and Hestrin, 2009). L2/3 commissural projecting IT neurons of the motor cortex project through the corpus callosum and other white matter (WM) commissures (e.g., the anterior commissure), forming axodendritic synapses at all cortical layers (yet dominating in L2/3) of the contralateral hemisphere (Lin et al., 2018; Tasic et al., 2018).

#### Extratelencephalic Projecting Neurons

Extratelencephalic projecting neurons, also known as the pyramidal tract neurons (PTN) in the M1, concentrate in L5b and contribute to the CST. In primates, the quantity of ET neurons is approximately eightfold lower than that of L2/3 IT neurons. With a more diffuse projection than the IT neurons, ET neurons target not only the telencephalon but also the ipsilateral subthalamic nucleus, brainstem, and spinal cord (Kita and Kita, 2012; Callaway et al., 2021). Importantly, ET neurons contain no interhemispheric projection (projection to the contralateral telencephalon), as proven by the retrograde tracing study by Reiner et al. (2003). However, in the rodent motor cortex, ET neurons received interhemispheric monosynaptic excitatory input from contralateral motor-related areas (with weak input from somatosensory areas), as reported in a retrograde labeling study (Mao et al., 2011; Hooks et al., 2013). While the input to L5 ET neurons in the human motor cortex is not clearly addressed, it is shown that ET neurons can be excited by receiving integrated signal from multiple IT neurons in both hemispheres, although the signal propagation is irreversible due to the nature of synapse connection. That is, whereas IT neurons can excite ET neurons, ET neurons cannot excite IT neurons in reverse. As a subset of L5 ET neurons, the anatomically and physiologically specialized Betz cells were also found in the premotor cortex (PM) and supplementary motor area (SMA) of the human brain with similar transcriptomic definition (Rivara et al., 2003; Bakken et al., 2021; Takata et al., 2021), consistent with the reported origin from the premotor cortex and the supplementary motor area of the CST (Rouiller et al., 1996; Seo and Jang, 2013). Collectively, L5 ET neurons in the motor cortex receive multiple inputs from both hemispheres and have a wide-range projection to cortical and subcortical areas. Additionally, L5 ET neurons in the motor-related areas (M1, PM, and SMA) share cellular similarity and may have close internal interactions so as to function as the cortical motor network.

#### Corticothalamic Neurons

Corticothalamic neurons project to the thalamus exclusively from L5a/L6a of the neocortex while connecting with IT neurons in the nearby lamina (i.e., L5a and L6a IT neurons) (Behrens et al., 2003; Kim et al., 2014; Yamawaki and Shepherd, 2015; Callaway et al., 2021). In the multiple circuit-analysis research by Yamawaki and Shepherd (2015), it was observed that the CT–IT connection presented only in L6, but not in L2/3. Although CT may interact with PTN through disynaptic circuitry involving an inhibitory IN, the direct connection of CT-PTN is almost absent, despite the vicinity of L6a, L5a, and L5b. However, in the mouse barrel cortex, CT neurons are reported to receive strong and focused innervation from L4 PN (Tanaka et al., 2011; Qi and Feldmeyer, 2016); however, whether this innervation exists in the motor cortex remains unknown. Moreover, despite the thalamus acting as a pivot of both the ascending and descending pathways and processing both afferent and efferent information (McFarland and Haber, 2002; Behrens et al., 2003), thalamocortical projections have almost no interaction with the projections of CT neurons at the ventrolateral nucleus (Yamawaki and Shepherd, 2015), consistent with the structural model of the cortico-thalamo-cortical loop (for a recent review, see Shepherd and Yamawaki, 2021). In terms of interhemispheric connectivity, while Molinari et al. (1985) reported bilateral components of the CT fibers from the motor cortex in rats and cats, a recent cell census proposed by the Brain Initiative Cell Census Network (BICCN) suggested that CT projections contain no interhemispheric components (Callaway et al., 2021). For interaction with INs, L6 CT neurons innervate local parvalbumin-expressing (PV^+^) and somatostatin-expressing (Sst^+^) INs, resulting in general inhibition in superficial layers (Baker et al., 2018). With a lower amount than IT neurons, the CT neurons with distinct neural properties are considered the third major subclass of neocortical principal neurons after IT and ET.

#### Near-Projecting and L6b Neurons

The L5/6 NP neurons and L6b neurons are mostly defined by transcriptomic signatures (Tasic et al., 2018; Langseth et al., 2021); their physiological properties are largely unknown. The L5/6 NP neurons, populating about 4% of the entire neocortical PNs, have no long-distance projections like the IT, ET, and CT neurons (Tasic et al., 2018; Bakken et al., 2021). Moreover, the L6b neurons (comprising 5% of total PN population) in the mouse visual cortex have been observed to send projections to the thalamus or anterior cingulate cortex (Tasic et al., 2018). However, there is no evidence reporting the physiology and function of the two subsets of principal neurons in the human neocortex owing to the difficulty in assessing activities in deep cortical layers *in vivo*. A viable investigation method analyzing L6b and NP neuron activity is needed to unveil their connectivity and physiological roles in the neocortical neural circuits.

### Neurotransmitter: γ-Aminobutyric Acid (GABA)

#### Parvalbumin-Expressing (PV^+^) Inhibitory Interneuron

The fast-spiking PV^+^ IN is the most common and well-studied neocortical inhibitory IN, distributed in L2–L6 of the neocortex (Bakken et al., 2021). PV^+^ INs stem from MGE, and can be morphologically divided into two subtypes: the basket cell (BC, greater quantity) and the chandelier cell (ChC, smaller quantity). Both BCs and ChCs have fast-spiking electrophysiological properties, yet the firing pattern (latency, frequency, etc.) differed between the two subtypes (Povysheva et al., 2013). For innervation target on the PN, it was reported that BCs targeted the cell soma and proximal dendrites, whereas ChCs innervated the axon initial segment (AIS) of the PN (Szentagothai and Arbib, 1974; Inan and Anderson, 2014).

Contrary to the common inhibitory effects elicited by BC, the postsynaptic effects elicited by ChCs are more intriguing. A series of rodent and human studies reported that ChCs received input from the PN of the ipsilateral and contralateral hemispheres (Lu et al., 2017). Woodruff et al. (2011) performed a patch-clamp study and reported that L2/3 ChCs were activated by the electrical stimulation of L1 (where most of the ChC dendrites reside), with different timing-dependent feedforward effects of both inhibition (ChC activation 5 ms prior to PN activation) and facilitation (ChC activation 15–30 ms prior to PN activation). Consistent with this study (Woodruff et al., 2011), ChCs were reported to excite postsynaptic PNs by depolarizing the AIS under certain postsynaptic membrane potential states (Szabadics et al., 2006; Woodruff et al., 2009, 2011), despite its classification as “GABAergic inhibitory IN.” In the developed neocortex, the existence of axo-axonic ChC provides an important strategy to optimize PN output *via* GABA_*A*_-receptor-induced excitation, as a specific GABA_*A*_ receptor (GABA_*A*_R-α2) is selectively enriched in the AIS of neocortical PNs (Gulledge and Stuart, 2003; Howard et al., 2005; Szabadics et al., 2006; Gao and Heldt, 2016).

Nevertheless, it should be noted that neocortical BCs stem from two different origins of CGE and MGE (Marin-Padilla, 1972; Butt et al., 2005; Gelman et al., 2011), with those from CGE expressing cholecystokinin (CCK), a neuropeptide of serotonin 3A Receptor (5HT_3*A*_R) family. PV^+^ BCs exceed CCK^+^ BCs in both quantity and size (Wang et al., 2002), and BCs are further classified as nest basket cells (mainly PV^+^), large basket cells (mainly PV^+^), and small basket cells (mainly CCK^+^) (for reviews, see Druga, 2009; Armstrong and Soltesz, 2012). Further description of CCK^+^ basket cells can be found in Section “Serotonin 3A Receptor-Expressing (5HT_3A_R^+^) Inhibitory Interneuron.”

#### Somatostatin-Expressing (Sst^+^) Inhibitory Interneuron

The second major GABAergic IN subclass (∼30% of the entire IN population) in the human motor cortex resides in L2–L6, expressing the neuropeptide somatostatin (Kowall and Beal, 1988; Wonders and Anderson, 2006; Bakken et al., 2021). Sst^+^ INs stem from MGE and predominantly consist of non-fast-spiking Martinotti cells and a minor proportion of non-Martinotti cells, with the latter demonstrating diverse morphological properties such as bitufted cell soma and long-range axonal projections (Rudy et al., 2011; Jiang et al., 2015; Tremblay et al., 2016; Urban-Ciecko and Barth, 2016).

The majority of Martinotti cells concentrates in L5 and L6, receiving excitatory synaptic input from L5 PN axon collaterals and forming synapses with other PN dendrites, and forming disynaptic inhibitory circuits between neighboring L5 PNs and translaminar recurrent inhibitory circuits targeting L2/3 IT neurons (Wang et al., 2004; Silberberg and Markram, 2007; Kätzel et al., 2011; Zolnik et al., 2020). In frequency-dependent disynaptic inhibition, it has been reported that fast (immediate) disynaptic inhibition was mediated by fast-spiking PV^+^ basket cells, whereas non-fast-spiking Martinotti cells mediated delayed disynaptic inhibition between L5 ET neurons (Silberberg and Markram, 2007; Berger et al., 2009; Obermayer et al., 2018).

In contrast to Sst^+^ Martinotti cells targeting L1, the quasi-fast spiking (fast-spiking pattern intermitted by random silences) Sst^+^ non-Martinotti cells targeting L4 were discovered over a century later than Martinotti cells (Ma et al., 2006; Xu et al., 2013). Of note, although the human M1 has been canonically characterized by the lack of the specific Layer 4, the existence of L4 neuron phenotypes in M1 (as in the middle temporal gyrus) was proven by the recent transcriptomic profiling study by BICCN (Bakken et al., 2021), aligning with the histological evidence from animals and humans (Cajal, 1899; Yamawaki et al., 2014; Barbas and García-Cabezas, 2015). Morphologically, non-Martinotti cells have ramified axons inside L4, and their dendritic projections do not extend to L1 (Muñoz et al., 2017; Scala et al., 2019). Thus, it is inferable that this type of non-Martinotti cells may coexist with L4-phenotype neurons scattered in L2/3 and L5 of the human M1. For synaptic connectivity, Sst^+^ non-Martinotti cells in L4 of mouse somatosensory cortex made more connections with PV^+^ INs than with PNs and this connection caused disinhibition of PV^+^ INs, controlling the processing of afferent input from the thalamus (Xu et al., 2013; Nigro et al., 2018). However, the specific role of non-Martinotti cells in the human motor cortex is not clear, as the presence of L4 in the motor cortex has not yet been firmly defined.

#### Serotonin 3A Receptor-Expressing (5HT_3*A*_R^+^) Inhibitory Interneuron

Apart from PV^+^ and Sst^+^ INs, almost all of the remaining 30% of cortical GABAergic INs express 5HT_3*A*_R, reside in the supragranular layers (L1–L3, especially abundant in L1) and specifically target PV^+^ and Sst^+^ INs in the neocortex (Chameau and van Hooft, 2006; Lee et al., 2010; Rudy et al., 2011; Bakken et al., 2021). 5HT_3*A*_R^+^ INs originate from CGE, and correspondingly, CGE-derived neurons specifically express 5HT_3*A*_R as the molecular marker (Wester and McBain, 2014; Wester et al., 2019). Similar to the heterogeneity of PV^+^ and Sst^+^ INs from MGE, CGE-derived INs also have highly diverse morphology and electrophysiological profiles (Lee et al., 2010; Miyoshi et al., 2010; Vucurovic et al., 2010). For example, in a genetic fate mapping study of CGE-derived INs by Miyoshi et al. (2010), nine types of morphologically and physiologically different INs were identified in the mice somatosensory cortex. In the same study (Miyoshi et al., 2010), it was also reported that around 75% of CGE-derived INs expressed reelin and vasoactive intestinal peptides (including calretinin and CCK, under the category of 5HT_3*A*_R), and had bipolar, single/double-bouquet, or multipolar morphology. The reelin-expressing INs were found to be late-spiking neurogliaform cells and INs with single bouquet or multipolar morphology, whereas INs expressing calretinin and CCK were burst-spiking double-bouquet or bipolar INs. While the double-bouquet cells mainly target fast-spiking PV^+^ BCs and ChCs (Hioki et al., 2018), single bouquet cells were reported to form synapses that selectively inhibited the activity of L2/3 INs, thus disinhibiting L5 ET neurons (Jiang et al., 2013). In particular, the multipolar CCK-expressing INs were classified as small basket cells, for its morphological and target resemblance with PV^+^ BCs (Armstrong and Soltesz, 2012; Goff and Goldberg, 2019). However, CCK^+^ BCs significantly differed from PV^+^ BCs by their high response sensitivity to acetylcholine, serotonin, or cannabinoids (Földy et al., 2007; Lee et al., 2010). Furthermore, CCK^+^ BCs had a non-fast-spiking profile with asynchronous GABA release onto the postsynaptic neurons, whereas PV^+^ BCs are fast-spiking cells with high GABA release synchronicity (Daw et al., 2009; Bartos and Elgueta, 2012).

### Hypothetical Neuron Landscape of Human M1

**FIGURE 2:**
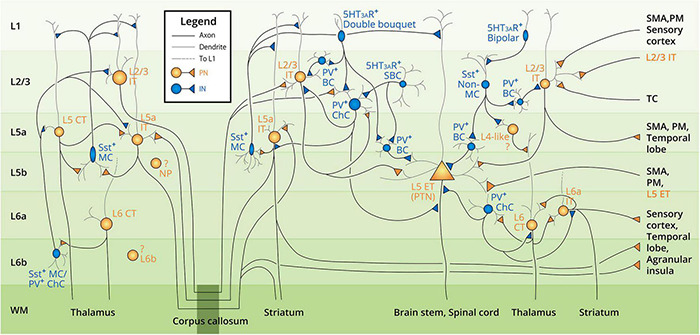
Summarized diagram of motor cortex neocortical neuron landscape and circuit connectivity. Neuron subtypes are labeled next to the cell soma from layer 1 to layer 6b (L1–L6b, labeled at the left side of the diagram), with the text color corresponding to the neuron type (orange–excitatory pyramidal neuron, PN; blue–inhibitory interneuron, IN). Gray lines denote the dendrites; black lines denote the axons. The target of the descending axons is indicated in the white matter (WM) area. Intracortical and interhemispheric connections are denoted by the division of the corpus callosum (dark green box at the bottom of the diagram). Note that all the neurons and connections are presented in both hemispheres. The question mark denotes the unclarified connectivity of the corresponding neurons. Abbreviations of the neuron subtypes can be found in Figure 1. Other abbreviations: MC, Martinotti cell; ChC, chandelier cell; BC, basket cell; SBC, small basket cell; PTN, pyramidal tract neuron; SMA, supplementary motor cortex; PM, premotor cortex; TC, thalamocortical axon.

Based on the reviewed literature of human and animal studies, we have summarized the neurocytological evidence into a schematic figure of the hypothetical neuronal landscape in the human motor cortex (Figure 2). As research evidence regarding the human motor cortex remains low in quantity, we therefore integrated the collective evidence from both animal and human studies that is included in the literature review of section 3 Cortical Neurons into Figure 2. The figure summarizes the motor cortex neuron subtypes, basic morphology, distribution in the six-layer structure, the intracortical and interhemispheric synaptic connection between neuron subtypes, and the evidenced communication pathways (input and output) with other cortical areas. All the displayed elements and relevant citations can be found in the text of section 3 Cortical Neurons.

## Neural Activities Involved in Transcranial Magnetic Stimulation

### Assessment of Transcranial Magnetic Stimulation-Related Neural Activities

The TMS-related neural activities can be measured at different levels along the descending of the efferent motor pathways (predominantly the CST). The neurophysiological measurements in most TMS and other non-invasive brain stimulation (NIBS) studies, as sorted by descending order along the CST, include (1) spinal cord-evoked potential (SCEP) at the cervical spinal cord level, (2) peristimulus time histogram (PSTH) from the single motor unit (SMU) recordings, and (3) motor-evoked potential (MEP) at the collective muscle output level.

Measurements along the descending pathways have revealed the different properties of TMS-related neural activities. Firstly, the transcranial electric stimulation (TES)-/TMS-evoked descending volleys recorded by epidural electrodes as the SCEP provide direct and valuable evidence of TMS-evoked general output from the brain (Patton and Amassian, 1954; Burke et al., 1993). Despite the invasiveness and subject limitation (patients with implanted epidural electrodes only) of the SCEP recordings, the discovery of direct and indirect waves (D-waves and I-waves) have illustrated the cortical network underlying TES/TMS in a robust dimension, inspiring numerous hypotheses and insights into the neural circuitries related to TMS and TES [for related SCEP reviews see Izumi (2001), Di Lazzaro et al. (2007, 2012, 2017), Di Lazzaro and Ziemann (2013), Di Lazzaro and Rothwell (2014), Ziemann, 2020]. Secondly, TMS-evoked SMU activity measured from a needle electrode inserted into the target muscle revealed the time-course spiking patterns in the measured motor unit as the PSTH. Compared with SCEP, less invasive SMU recordings can be obtained from normal subjects. Similarly, PSTH also clearly showed the arrival of D-wave and I-waves in a spatiotemporal order, similar to SCEP (Day et al., 1989). Finally, based on surface electromyography (EMG), MEP reflected the integrity and excitability of the entire motor cortex and pathways. Up to now, the majority of TMS outcomes have been measured by MEP, both in studies unveiling the mechanisms of TMS or estimating NIBS treatment effects in neurology patients. Overall, the measurement at all levels along the CST has offered complementary experimental evidence regarding the property and pattern of TMS- and TES-induced physiological process, which is largely reflected in the selectivity of different TMS pulse properties and paradigms.

### Transcranial Magnetic Stimulation Pulse Property Selectivity

Altering the intrinsic properties (phasic pattern, pulse width, pulse intensity, induced current direction, etc.) of a TMS pulse resulted in outcome difference, indicating the selectivity of the underlying neural circuits to the pulse. For phasic patterns, the resting and active motor thresholds (RMT and AMT) were higher when assessed with monophasic than biphasic TMS pulses (all with a figure-of-eight coil, unless otherwise specified) [Di Lazzaro et al., 2001a; Kammer et al., 2001; Sommer et al., 2006, 2018; see also Casula et al. (2018) for contradictory results]. Notably, the optimal coil orientations contradicted each other in the monophasic and biphasic TMS pulses. In monophasic TMS, MT was lower when the induced current followed the posterior–anterior (PA) direction than the anterior–posterior (AP) direction, whereas in biphasic TMS, the MT was lower when the current flow was in the AP–PA direction (Di Lazzaro et al., 2001a; Sommer et al., 2006, 2018). Along with lower MT, biphasic TMS also yielded shorter MEP latency than monophasic TMS (Sommer et al., 2006), indicating the higher efficacy of biphasic TMS pulse in depolarizing neurons. The difference between monophasic and biphasic TMS was further supported by SCEP and EEG studies showing that the cortical origin of this difference was the neural components with different excitation threshold resulted in the response difference between monophasic and biphasic TMS (Di Lazzaro et al., 2001a; Casula et al., 2018). For pulse width, it has been shown that lengthening the pulse width decreased MT and MEP latency (Peterchev et al., 2008; D’Ostilio et al., 2016; Casula et al., 2018). Combined with coil orientation, D’Ostilio et al. (2016) and Hannah and Rothwell (2017) reported that AP-TMS with shorter pulse width (30 μs, 60 μs) resulted in longer onset latency in both MEP and SMU (PSTH) responses in comparison to a 120-μs pulse, yet no specific difference was observed in PA-TMS. For pulse intensity, SCEP recordings indicated that subthreshold pulses may have an impact on the intracortical circuits (Di Lazzaro et al., 1998b), which in subsequently became the theoretical basis of the well-known short interval intracortical inhibition (SICI) paradigm (Kujirai et al., 1993; Izumi, 2001; see Section “M1 Paired-Pulse Transcranial Magnetic Stimulation”). Moreover, given that the TMS stimulation depth in the cortex is correlated with pulse intensity, we speculate that the difference between AMT and RMT-TMS may not merely result from motor drive but also subject to the stimulation depth of the pulse. However, as it remains difficult to investigate the influences of the motor drive independently, we therefore do not make further speculations on the impact of AMT-TMS. Finally, in terms of current direction, the reason that the TMS-evoked descending volleys differed significantly when altering current direction remains enigmatic [for recent reviews, see Ziemann (2020), Opie and Semmler (2021)]. Until now, studies measuring SCEP, PSTH, and MEP (Day et al., 1989; Sakai et al., 1997; Di Lazzaro et al., 1998a,b, 2001a,2001b,^2004^; Ni et al., 2011a) have demonstrated that (1) lateromedial (LM)-TMS recruited D-waves at the lowest intensity and had the shortest MEP/PSTH onset latency among all directions; (2) PA-TMS recruited both early and late I-waves at a lower intensity while D-waves emerged as the intensity increased, with a 1-ms MEP/PSTH onset delay compared to LM-TMS; and (3) AP-TMS showed distinct recruitment of late I-waves in SCEP (which may differ from that evoked by PA-TMS in neural origin), had the highest threshold, the longest MEP/PSTH onset latency, and more functional relevance. Overall, the most agreed opinion on the TMS current direction is that varying coil orientation can induce a remarkable change in the recruited neural components (neurons, synapses, axons), and can thus alter all aspects of the outcome. However, it should be noted that the I-waves recruited by different TMS forms (phasic pattern, pulse width, pulse intensity, and current direction) may originate from different neuro populations of the motor cortex, as evidence exists that the SCEP recordings of monophasic PA- and AP-TMS, as well as by biphasic PA-AP pulse show difference in latency and frequency of the I-waves (Di Lazzaro et al., 2017). Specifically, monophasic PA-TMS preferentially evokes an I1-wave at threshold intensity, while AP-TMS preferentially recruits I-waves with lower frequency and longer latency. Although PA-TMS at high intensity also evokes later I-waves, the neural population activated by the two pulses is thought to be different, despite the possibility that these circuits may overlap.

### Transcranial Magnetic Stimulation Paradigm Selectivity

**FIGURE 3:**
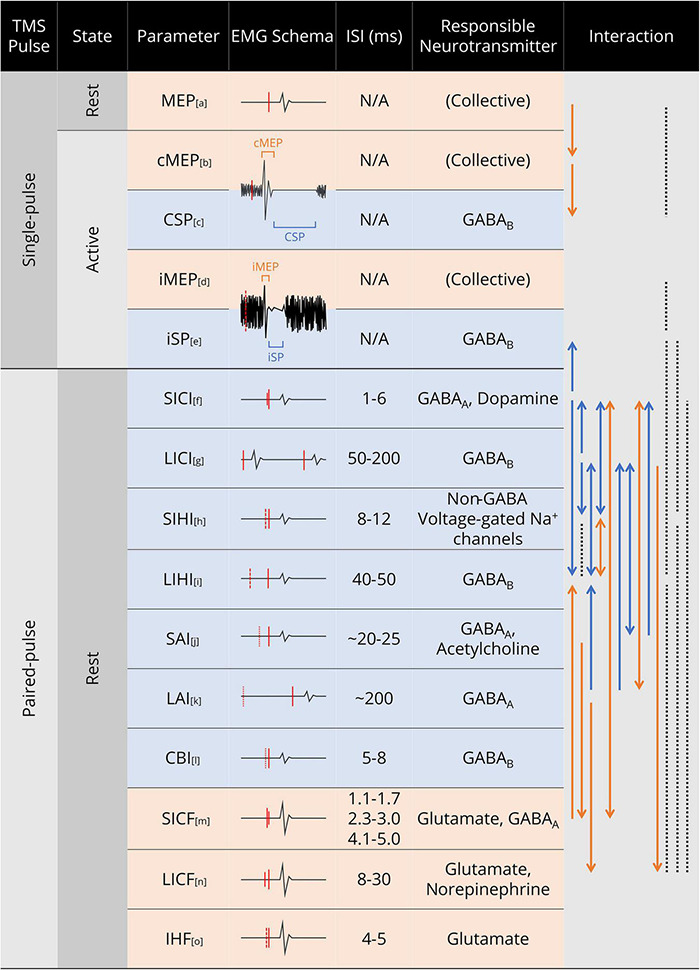
Summary of common TMS paradigms and parameters. In the EMG Schema column, red solid line denotes TMS pulse; red dashed line denotes TMS pulse in the contralateral hemisphere, with the line length indicating stimulus intensity; red dotted line denotes median nerve electric stimulus in SAI and long interval afferent inhibition (LAI), and cerebellar stimulus in CBI. In the Interaction column, solid line denotes the relationship between the two parameters at both ends; dotted line denotes no interaction/correlation was observed between the two parameters; arrow denotes the direction of the interaction (facilitation/inhibition); for line colors, orange denotes facilitation and blue denotes inhibition. Abbreviations: cMEP, contralateral MEP; CSP, cortical silent period; iMEP, ipsilateral MEP; iSP, ipsilateral silent period; SICI, short interval intracortical inhibition; LICI, long interval intracortical inhibition; SIHI, short interval interhemispheric inhibition; LIHI, long interval interhemispheric inhibition; SAI, short latency afferent inhibition; LAI, long latency afferent inhibition; CBI, cerebellar inhibition; SICF, short interval intracortical facilitation; LICF, long interval intracortical facilitation; IHF, interhemispheric facilitation. References for the paradigms and interactions: a. (Izumi, 2001; Rossini et al., 2015); b. (Ngomo et al., 2012); c. (Orth and Rothwell, 2004); d. (Wassermann et al., 1994; Tazoe and Perez, 2014); e. (Chen et al., 2003; Jung et al., 2006); f. (Chen, 2004; Trompetto et al., 2004; Lee et al., 2007; Ni et al., 2007); g. (Daskalakis et al., 2002; Lee et al., 2007; Udupa et al., 2009); h. (Daskalakis et al., 2002; Irlbacher et al., 2007; Ghosh et al., 2013); i. (Chen, 2004; Lee et al., 2007; Udupa et al., 2010); j. (Udupa et al., 2009; Chen, 2017; Saravanamuttu et al., 2021); k. (Trompetto et al., 2001; Sailer et al., 2002; Kobayashi et al., 2003); l. (Fernandez et al., 2018; Hassan et al., 2019); m. (Avanzino et al., 2007; Chen, 2017; Qasem et al., 2020); n. (Kobayashi et al., 2003; Chen, 2004; Lee et al., 2007; Udupa et al., 2010); o. (Hanajima et al., 2001; Baumer et al., 2006; Belyk et al., 2021).

Based on the experimental evidence, we present a comprehensive schematic of TMS paradigm selectivity in Figure 3, showing the phenomenal properties, mediating neurotransmitter, and interactions of the common TMS paradigms. As the paradigm selectivity is mostly reflected in paired-pulse TMS, we have divided the paired-pulse TMS experimental evidence according to the stimulation site and discussed the selectivity of M1 paired-pulse TMS and motor network dual-site TMS, respectively.

#### M1 Paired-Pulse Transcranial Magnetic Stimulation

M1 paired-pulse TMS paradigms have been widely used to investigate cortical facilitation and inhibition. Although studies using multipulse paradigms have also been proposed, the basic strategy of multipulse protocols can be regarded as an integration of two or more single- and paired-pulse paradigms. Supported by pharmacological evidence [for reviews, see Ziemann et al. (2015), Darmani and Ziemann (2019)], it has been widely accepted that different TMS paradigms preferentially activate different excitatory/inhibitory circuits (Figure 3). As cortical inhibition has been extensively investigated and the results are highly systematic, in the present review, we summarize the patterns and interaction of cortical inhibition in Figure 3, and we specifically focus the discussion on cortical facilitation phenomena, which, at present, remain poorly understood.

As presented in Figure 3, cortical inhibition has been extensively investigated and evidenced to have relationship with cortical GABA activity, which can alternatively be assessed non-invasively using magnetic resonance spectroscopy (MRS). Specifically, the prior study that connected TMS and MRS measurements have revealed that SICI at 1-ms ISI was negatively correlated with GABA level in the sensorimotor cortex (SMC GABA level) while SICI at 2.5-ms ISI did not show specific correlation (Stagg et al., 2011). Accordingly, the authors speculated that the 1-ms SICI and SMC GABA level correlation may reflect extra-synaptic GABA tone, which is different from those involved in 2.5-ms SICI. A subsequent study that investigated the comprehensive relationship between TMS-measured SICI, LICI, and CSP supported the previous results, showing no relationship between 3-ms SICI, 100-ms LICI, and SMC GABA level (Tremblay et al., 2012). Tremblay et al. also reported a moderate positive correlation between CSP duration and SMC glutamate + glutamine level, as well as a positive correlation of SMC GABA and glutamate levels. Based on the prior pharmaceutical evidence that the 2–4-ms SICI may reflect low-threshold GABA_*A*_ receptor activity (Ilic et al., 2002), it can therefore be inferred that the mechanisms of 1-ms SICI and 2–4-ms SICI may also come from different ways that GABA acts upon the neurons (i.e., extra-synaptic tone or GABA receptor activity), even though the targeting neurons may be the same. However, contrasting results emerged from recent studies investigating the effect of aging. Hermans et al. (2018) reported reduction of 1- and 3-ms SICI in the group of older adults with no alteration of SMC GABA level compared with younger adults, along with no correlation observed between SICI and SMC GABA level in both groups. Additionally, another study reported reduced SMC GABA level in older adults compared with younger population, along with the task-related SICI (3-ms ISI) reduction of the dominant hemisphere. Similarly, no correlation was found between SICI and MRS outcomes (Cuypers et al., 2020). Regarding the disconnection between MRS-measured SMC GABA level and 2–4-ms SICI outcomes, two reasons can be considered. Firstly, as the MRS measures the general GABA level of the sensorimotor cortex due to the minimum of a 3 × 3 × 3-cm voxel size to acquire reliable outcome (Mullins et al., 2014), possibility exists that the GABA level from the somatosensory cortex (included in MRS outcomes) may affect the MRS-TMS correlation while the paired-pulse TMS reflects mainly the GABA activity in the M1. Secondly, since TMS is an external magnetic stimulation measured through the CST output (MEP, SCEP, etc., reflecting merely the CST-related pathway GABA activity) and MRS outcomes reflect a general GABA level within the SMC, the correlation can be blurred by the possibility that MRS outcomes may come from circuits and neural populations that are not involved in TMS.

Apart from the inhibitory neural circuits, excitatory circuits give rise to paired-pulse cortical facilitation phenomena, which can be subdivided regarding the paired-pulse interstimulus interval (ISI) into (1) short interval intracortical facilitation (SICF), (2) long interval intracortical facilitation (LICF), and (3) interhemispheric facilitation (IHF) (Figure 3). For SICF, it is reported that the ISI with peak MEP facilitation was consistent with the timings of the I-wave; thus, SICF has been used widely as a non-invasive method to evaluate I-wave recruitment, acting as a substitute for invasive SCEP and SMU recordings (Tokimura et al., 1996; Ziemann et al., 1998; Hannah and Rothwell, 2017; Van den Bos et al., 2018; Ziemann, 2020). However, the controversial results from the SICF recruitment curve (the curve plot of SICF-MEP amplitude in different ISIs) in the AP and PA directions shed brought to light some uncertainty in the correspondence between I-waves and the SICF curve. Specifically, the SICF curve assessed by AP-TMS showed enhanced I1-wave-latency facilitation, whereas PA-TMS yielded comparable facilitation for all I-wave latencies (Delvendahl et al., 2014), contradictory to the SCEP evidence showing preferential late I-wave recruitment by AP-TMS. Meanwhile, a recent study reported the facilitation of SICF by the presence of short interval afferent inhibition (SAI) (Saravanamuttu et al., 2021), which also contradicted the PSTH evidence showing inhibition of late I-waves (especially late I-waves in PA-TMS) by SAI (Ni et al., 2011a). Consequently, it may be problematic to analogize SICF to I-waves, because the causal link between SICF and I-wave recruitment is merely the timing consistency, which is inadequate for equalizing the two physiological processes. For LICF, the origin and underlying circuitry remains unknown, despite its early discovery in 1993 (Kujirai et al., 1993) and broad application in basic and clinical TMS studies. One of the complex conundrums regarding LICF is that almost all paradigm-interaction studies found no interaction of LICF with other TMS paradigms (Chen, 2004; Lee et al., 2007; Reis et al., 2008; Udupa et al., 2010; Ni et al., 2011c), not even corticospinal descending volleys in SCEP recordings (Ni et al., 2011b). However, an early study (Nakamura et al., 1997) reported late I-wave facilitation in the LICF paradigm, and another two (Kobayashi et al., 2003; Ni et al., 2007) reported LICF facilitation by long interval intracortical inhibition (LICI) and long interval afferent inhibition (LAI). As the cortical origin of LICF has long been known to be different from those generating the high-frequency I-waves (reflected in the SCEP) (Kujirai et al., 1993; Nakamura et al., 1997; Di Lazzaro et al., 2006), it is therefore inferable that LICF was facilitated by LICI mechanisms (upon which LICI and LAI act), possibly due to selective disinhibition of the silenced PNs by LICI. For IHF, little evidence has been provided since the report of M1-IHF (hand motor area) existence by paired-pulse TMS at an ISI of 4–5 ms (Ferbert et al., 1992; Ugawa et al., 1993; Hanajima et al., 2001). Although Sommer et al. (2012) observed inconsistent M1-IHF at 2 ms ISI paired-pulse TMS that was diminished by sodium channel blocker carbamazepine, the 2 ms ISI brought uncertainty about the observed IHF as it was significantly lower than common M1–M1 interhemispheric transfer time by TMS (8.8–12.2 ms) (Cracco et al., 1989). As IHF is thought to be masked by massive cortical inhibitory mechanisms (especially the powerful IHI), the investigation into IHF appears unviable due to its inconsistency and vulnerability to IHI in paired-pulse TMS paradigms. However, it should be noted that IHF is a phenomenon actually present in cortical activities, as the excitatory attribute of callosal fibers has long been proved (Kawaguchi, 1992; Conti and Manzoni, 1994). That is, excitatory callosal axons project to local inhibitory INs, generating IHI, which, as an alternative can be interpreted as a structure with “superficial IHI above deeper callosal excitation.” Accordingly, if the “superficial” inhibitory effects could be reduced or eliminated, IHF presence may therefore be expected and observed. This insight has gradually gathered interest, and researches attempting to measure online and offline IHF emerged in recent years. In a recent study by Belyk et al. (2021) using paired-pulse IHF with 10 and 50 ms ISI, the authors reported paradoxical facilitation presenting with increased conditioning stimulus (CS) intensity; therefore, it was suggested that when CS is sufficiently intensive, it may overwhelm the contralateral local inhibitory circuits and eventually demonstrate IHF in the common IHI paradigm. Together, we suggest that the previous consideration of “difficult to investigate” for IHF has now reached the prime stage for investigation, to determine the expected clinical application perspectives of IHF in stroke, brain injury, neurodegenerative diseases, and compensation of impaired brain function.

#### Motor Network Dual-Site Transcranial Magnetic Stimulation

In humans, the cortical motor network consists of M1, dorsal and ventral PM (PMd and PMv), SMA, cingulate motor area, somatosensory cortex (S1), and the inferior parietal lobule (IPL) (Volz et al., 2015; Ruddy et al., 2017). Unlike the M1 paired-pulse paradigm selectivity, dual-site TMS targeting M1 and non-M1 motor areas yielded highly stimulus intensity- and ISI-dependent results. For the PM-M1 interaction, the first report by Civardi et al. (2001) using dual-site TMS and PSTH demonstrated that the motor response of M1-test stimulus (TS) was inhibited by a preceding PMd-CS (90% AMT) at 6 ms ISI, and that when the CS intensity was increased to 110–120% AMT, the effect became facilitatory. Subsequently, MEP of M1-TS was also reported to be facilitated by a delayed ipsilateral suprathreshold PMd-CS at 1.2 and 4.4 ms ISI in humans, yet the facilitation at 1.2 ms ISI was abolished during muscle activation (note that the CS intensity, ISI timing, and discrimination in muscle activation resembled that of M1-SICF) (Ortu et al., 2008; Groppa et al., 2012a,b). For interhemispheric interaction, Baumer et al. (2006) reported IHF effects by conditioning the contralateral PMd with a subthreshold pulse of 60/80% AMT at 6/8 ms ISI, respectively. Similar to the ipsilateral PMd-M1 interaction, the IHF effect also turned into IHI when the CS intensity was set to 90–110% RMT and applied prior to the TS by an 8–10 ms ISI (Mochizuki et al., 2004). Based on this basal intracortical facilitatory and interhemispheric inhibitory effect of suprathreshold PMd-TMS, a further triple-pulse TMS study by Koch et al. (2007) revealed that conditioning the left PMd with paired-pulse TMS also altered the outcome of this basal facilitation and inhibition that PMd exerted on ipsilateral and contralateral M1. Specifically, the pairing of an 80–100% (but not 70%) RMT PMd-CS1 and a 110% PMd-CS2 by a 5 ms ISI canceled the 6 ms PMd-ipsilateral M1 facilitation (i.e., the M1-TS was applied 6 ms after CS2), as well as the 8 ms PMd-contralateral M1 inhibition at both 1 ms and 5 ms CS1-CS2 ISI. Contrary to the basal intracortical facilitatory and interhemispheric inhibitory effects that PMd exerts on M1, PMv tends to elicit more intracortical inhibitory and interhemispheric facilitatory effects on M1 than PMd, as proven by intracortical microstimulation (ICMS) study (Tokuno and Nambu, 2000; Côté et al., 2017), which suggests a substantial functional difference between PMv and PMd. Correspondingly, as reported in rest-state dual-site TMS studies, conditioning ipsilateral PMv 8 ms (80% RMT) or contralateral PMv 40 ms (90 and 110% RMT) before M1-TS led to MEP inhibition (Buch et al., 2011; Côté et al., 2017; Fiori et al., 2017). For the SMA-M1 interaction, the glutamatergic connectivity between M1 and SMA has long been proven in primate and human studies (Tokuno and Nambu, 2000). In humans, this excitatory connectivity typically underpins bimanual coordination, action preparation, and motor imagery (Al-Wasity et al., 2021; Neige et al., 2021). Using dual-site TMS, the SMA-M1 facilitation was confirmed that SMA-CS (140% AMT) applied 6/7/8 ms prior to M1-TMS induced stable rest MEP facilitation at all ISIs in younger adults but not in older adults (Rurak et al., 2021a). However, another study also adopted 140% AMT SMA-CS and a subsequent M1-TS by 6/8 ms ISI, but the MEP facilitation was observed only in younger adults with 6 ms ISI; older adults showed no facilitation at any ISIs (Green et al., 2018). Overall, the experimental evidence for motor network dual-site TMS suggests a close circuit wiring inside the motor network, although the results depend highly on the dual-site TMS paradigm even when assessed in the rest state. As the task-related motor network connectivity and interaction may be much more complicated due to the huge diversity of task types, we do not perform further review of the task-dependent motor network connectivity. Nevertheless, possible reasons for this varying evidence can be summarized as (1) TMS coil size limitation and (2) results contamination by direct CST projections from the non-M1 motor areas. In (1), except for two PMd-M1 dual-site TMS studies (Groppa et al., 2012a,b) in which a customized high-focal TMS coil was used and one S1-M1 dual-site TMS study (Brown et al., 2019) that used figure-of-eight coils with an inner diameter of 25 mm, all other aforementioned dual-site TMS studies targeting M1/PM/SMA adopted a figure-of-eight coil with ≥50 mm loop diameter to perform the non-M1 TMS. Therefore, it is technically difficult to simultaneously aim M1 and ipsilateral nearby areas with two 50/70 mm coils, because the PM-M1 and SMA-M1 distances are both less than 30 mm, and even less than 20 mm in the case of ipsilateral M1-S1 (Mochizuki et al., 2004; Holmes and Tamè, 2018; Brown et al., 2019; Rurak et al., 2021b). For reason (2), while the fibers with largest diameter and fastest conducting velocity in CST come from M1, it was also reported that the CST contained direct projections from SMA, PMd, parietal cortex (mainly terminated in the brain stem), S1, and PMv, presented in descending order of the axon conduction velocity (Firmin et al., 2014; Innocenti et al., 2019). Accordingly, we may expect the change in M1-MEP to be a result of CST output change from other cortical areas instead of M1 excitability. As the axon conducting velocities from different cortical areas vary from 6.01 to 15.49 m/s in the monkey CST (Innocenti et al., 2019) and this variability may result in the iteration of corticospinal descending volleys, future studies are expected to examine the descending volley modulation under motor network dual-site TMS protocols.

## Discussion: Connecting System and Cellular Process Underlying Transcranial Magnetic Stimulation

In this chapter, we integrate the neurophysiological evidence based on the presented neuron landscape (Figure 2) and propose our model of the underlying neural mechanisms of motor cortex TMS. Based on our literature review, we connect the TMS results and cellular/anatomic evidence from the perspectives of (1) stimulus intensity, (2) paired-pulse timing, and (3) TMS-induced current direction.

### Paired-Pulse Timing

**FIGURE 4:**
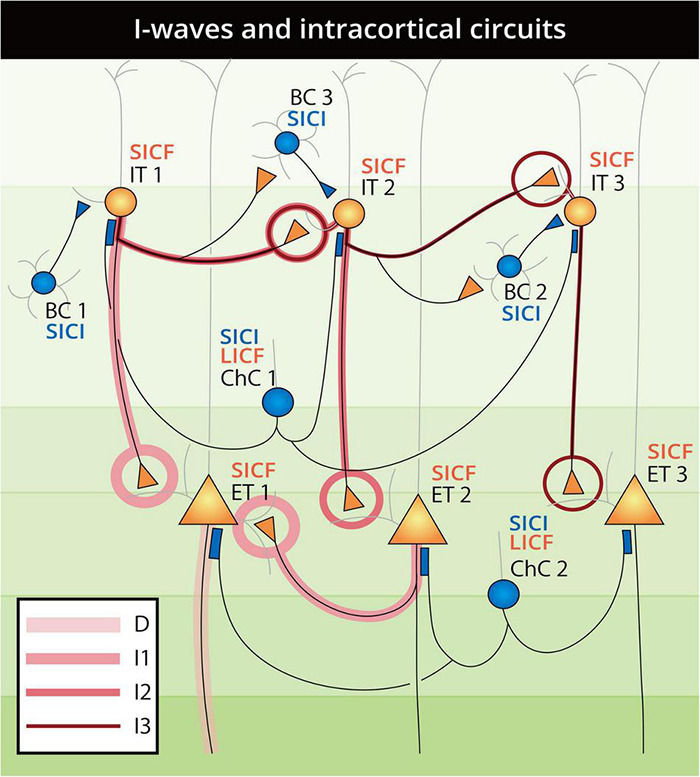
Schematic landscape of D-wave and I-wave generation and paired-pulse SICI, SICF, and LICF intracortical circuits. Neurons are constructed and labeled as in Figure 2 and are numbered to illustrate the pathways of D-waves and I-waves. D-waves arise from the direct firing of ET neurons, as lined from the axon of ET1. The I1-wave is generated through the IT1-ET1 and ET2-ET1 types of circuit with one synaptic connection, which accounts for the ∼1.5 ms lag after the D-wave. Accordingly, the I2-wave is generated through the IT1-IT2-ET2 type of circuit with two synaptic connections, and the I3-wave is generated from the IT1-IT2-IT3-ET3 pathway with three synaptic connections. As the suprathreshold TMS pulse activates all neurons at the same time, the arrival of BC1 inhibition to the IT1 neuron lags one synaptic delay after the first firing of the IT1 neuron (generating the I1-wave), and can therefore explain the SCEP evidence of no inhibition of the I1-wave in the inhibitory protocols. Subsequently, with the regulation of INs, the I2- and later I-waves can be selectively suppressed by a timing-matched subthreshold pulse and displayed as SICI. When the influence of ChC at the AIS turned from inhibition to disinhibition (excitation) in the 8–30 ms time window, the outcome shifts consecutively from inhibition (SICI) to facilitation (LICF). As SICF selectively recruits the residual EPSPs from the unfired IT and ET neurons at a short latency, the regulation of INs may be eliminated by the second pulse arriving at the firing interval of the highly synchronous fast-spiking BCs and ChCs.

The effect of intracortical and interhemispheric paired-pulse TMS (see Section “Transcranial Magnetic Stimulation Paradigm Selectivity” and Figure 3) can be expected to reflect certain neuron connectivity through precise ISI and pulse intensity. In chemical synapses, the latency from the peak of presynaptic action potential (AP) to the initiation of postsynaptic EPSP between PNs was approximately 1.5 ms, and the same latency was preserved in both IN–IN and IN–PN synaptic connections (Di Lazzaro et al., 1999; Guo et al., 2012). From the consistency of synaptic latency and SICF timing, the contribution of a translaminar PN–PN synaptic chain in SICF can be expected. That is, whereas the first suprathreshold pulse generates EPSPs in most PNs, only a part of them fires, in which the generated EPSP is strong enough to depolarize the membrane to the AP threshold. The “residual” EPSPs in the unfired PNs can then be raised above the threshold by the upcoming subthreshold stimulus, generating additional APs, which is reflected as MEP facilitation and late I-wave facilitation in SCEP recordings (Di Lazzaro et al., 1999; Ziemann, 2020). The facilitation in SICF may circumvent the inhibitory INs, as there is abundant evidence to show that with ISI at the SICF curve troughs (indicating the refractory period of the fired PN) and even under pharmaceutical control, MEP remained facilitated in the SICF paradigm (Ziemann et al., 1998; Delvendahl et al., 2014; Van den Bos et al., 2018; Opie and Semmler, 2021). The facilitation of SICF may come from both M1 and PMd through direct synaptic connection with L2/3 IT neurons targeting L5 PN. However, as discussed in Section “M1 Paired-Pulse Transcranial Magnetic Stimulation,” it should be noted that as SICF and I-wave are not completely identical, the suppression of I-waves by inhibitory protocols (Di Lazzaro et al., 1998b; Ni et al., 2011b) can therefore involve an IN component, which is different from that of SICF. Specifically, the generation of I-waves [for a comprehensive review, see Ziemann (2020)] represents an aggregative output of all cortical neurons excited by a TMS pulse, whereas SICF may selectively recruit the EPSPs in the PNs by the paired-pulse (Figure 4). In paired-pulse inhibitory protocols, such as SICI, LICI, and IHI, the involvement of cortical INs can also be analyzed in the pulse timing. SICI and LICF were first reported by Kujirai et al. (1993) with constant CS and TS intensity and varied ISI, in which a consecutive evolution from inhibition to disinhibition was revealed as having ISI as the sole variable. By analogy of the timing and pulse intensity, we speculated that the two phenomena may be related to similar neural circuits involving cortical BC and ChC function [see Section “Parvalbumin-Expressing (PV^+^) Inhibitory Interneuron”]. There are three reasons for this speculation. First, in both SICI and LICF, the first pulse is subthreshold (usually 60–90% AMT), which cannot reach the deeper layers as threshold pulse does. Accordingly, the subthreshold pulse results in the excitation of the supragranular PV^+^ BC and ChC, which can elicit powerful and extensive feedforward influence onto the incoming second suprathreshold pulse. Second, in addition to the demonstrated contribution of fast-spiking BC in SICI generation (Aberra et al., 2020; Fong et al., 2021), the timings of SICI and LICF (1–6 ms and 8–30 ms) were also consistent with the time window of ChC inhibition and disinhibition in patch-clamp studies (Woodruff et al., 2011). Third, it has been proved that ChCs mainly innervate the AIS of PN through GABA_*A*_R-α2 (Gulledge and Stuart, 2003; Howard et al., 2005; Szabadics et al., 2006; Gao and Heldt, 2016). As pharmaceutical studies have reported the effect of GABA_*A*_R (especially α2 or α3 subunits) in SICI (Kujirai et al., 1993; Di Lazzaro et al., 2007), there is the possibility that SICI and LICF are related to supragranular ChC activity. For LICI (CSP) and LAI that have an extremely long latency of 50–200 ms, we propose that the asynchronous GABA release from a different subset of INs may be a contributing factor (Hefft and Jonas, 2005). In the neocortex, this asynchrony was mainly produced by Sst^+^ Martinotti cells [the regulator of neocortical surround inhibition, see Section “Somatostatin-Expressing (Sst^+^) Inhibitory Interneuron”], causing persisting inhibition lasting 42.2–210.3 ms after presynaptic PN firing (Deng et al., 2020). Additionally, in the amygdala, neocortical CCK^+^ BCs were also reported to delay PN firing by up to 40 ms (Veres et al., 2017). Although whether this long-time delay in the amygdala is preserved in the neocortex remains unclear, neocortical Sst^+^ and 5HT_3*A*_R^+^ INs that cause GABA release asynchrony and delay may be related to the mechanisms of the long latency paired-pulse TMS paradigms with ISI > 50 ms.

However, as there is evidence that dopamine, acetylcholine, and norepinephrine can also affect the activity of INs and PNs apart from the main neurotransmitter of glutamate and GABA, and that regulating those neurotransmitters actually alters the TMS outcome (Ziemann et al., 2015; Turco et al., 2018), questions regarding the neural substrates of TMS remain unanswered. It is also necessary to take other neurotransmitters into account when explaining the TMS mechanisms in the future.

### Transcranial Magnetic Stimulation-Induced Current Direction

For TMS current directions, we believe that further insight can be obtained by zooming out from the cellular structure to the gross anatomy of the precentral gyrus. Even with the “focal” figure-of-eight coil, the excitation area of M1-TMS can cover both the anterior and posterior banks of the precentral gyrus, with strongest electric field presenting at the gyral crown (Aberra et al., 2020). In this case, the side in which the axon direction follows the induced current can be preferentially excited compared with the opposite side. That is, AP- and PA-TMS can result in preferential excitation of the anterior and posterior bank of precentral gyrus, respectively. This preference was partly reflected in computation models, in which the threshold of the anterior bank was lower than the posterior bank in AP-TMS than in PA-TMS, and vice versa (Aberra et al., 2020; Wendt et al., 2021). In addition, the most extensively investigated M1 area—the “hand knob,” has been strongly evidenced as an area mainly located in the posterior bank of the precentral gyrus, with the opposing anterior bank being largely PMd, which has a high neuroanatomical variability across individuals (Yousry et al., 1997; Siebner, 2020; Eichert et al., 2021; Simone et al., 2021). We highlight this evidence because it implies a potential explanation for D-wave and I-wave recruitment that can fit almost all experimental evidence (I-wave recruitment, MEP latency, inter-individual differences etc.; for details, see Sections “Transcranial Magnetic Stimulation Pulse Property Selectivity,” “Motor Network Dual-Site Transcranial Magnetic Stimulation”). Specifically, we propose the hypothesis that AP- and PA-TMS can represent the anterior–posterior or posterior–anterior bank preferential excitation order of the precentral gyrus, whereas LM-TMS excites both the banks of the precentral gyrus to the same extent, resulting in lowest threshold and shortest MEP latency. For I-wave preferential induction by PA- and AP-TMS, we speculate that the late I-wave selectivity of AP-TMS may be related to the IT neurons located in the anterior bank of the precentral gyrus, which can be priorly excited by AP-TMS. These IT neurons may receive the regulation of INs by disynaptic inhibition during the synaptic relay from the anterior bank to the “hand knob” M1 ET neurons, resulting in the late I-wave inhibition in the SAI, SICI, and LICI (Di Lazzaro et al., 1999; Ilic et al., 2002; Ni et al., 2011b) paradigms (see Section “M1 Paired-Pulse Transcranial Magnetic Stimulation”).

## Implications and Future Directions

Efforts to establish the connection of the microstructure and macro-outcome of the human brain are in a preliminary stage, not only for TMS but in all aspects of neurocytology. Based on our literature review, we identified a number of research gaps underlying TMS, both in cellular and system neurocytology. First, we noticed that the major cellular evidence on neocortical neurons comes from animal studies of various cortical areas, which is influenced by the differences in species and cerebral areas when we seek to explain the specific mechanisms of human motor cortex TMS. However, we acknowledge the technical and ethical conundrums of conducting human cellular experiments (even on postmortem tissue). Consequently, alternative methods for analyzing the interspecies differences have emerged [e.g., the BICCN studies on the motor cortex (Bakken et al., 2021; Kalmbach et al., 2021)], which is a helpful solution to the understanding of the human brain from animal experiments. Second, even though TMS has been practiced for over 35 years, a large potential for development remains. To perform more precise, detailed investigations, as well more efficient neuromodulations, practical modifications such as a more focal TMS coil design, a narrower TMS pulse width, and a reduction in TMS coil size are expected in the future. Third, since the final goal is to “decipher and optimize the human brain,” it is ultimately inevitable that we should link the highly developed cellular neuroscience knowledge with system neuroscience outcomes, so that precise and efficient neuromodulation based on this connection can be achieved in clinical practice.

Consequently, at the preliminary stage, we begin with the most unified, most fundamental, and simplest analysis in M1-TMS at rest or with simple movement. Based on this analysis, future investigations can be expected that integrate complex movements, a combination of brain areas, and neuroplasticity protocols. While we acknowledge that we cannot fully answer the questions we posed in the introduction, which are important research gaps we have identified, we intend to leave these questions open, in the hope that further investigators can be inspired and make novel discoveries based on the existing evidence and hypothetical models.

## Conclusion

In this integrative review, we have reviewed the existing literature related to motor cortex neuron biology and TMS; on this basis, we have proposed a preliminary model for the micro–macro connections between neocortical neurons and TMS in the human motor cortex. Additionally, for the unanswered questions underlying certain TMS paradigms (such as the mechanisms of LICF, IHF, and the TMS-induced current selectivity on neural circuits), we provided hypotheses based on the reviewed literature. As a preliminary endeavor to bridge the microscopic and macroscopic facts regarding TMS, the present integrative review provides a foundation for future investigations, as well as a platform for technical advances and applications of TMS.

## Author Contributions

DT organized the literature database, performed the review, and wrote the first draft of the manuscript. S-II provided revision of the manuscript. Both authors contributed to conception and design of the study, manuscript revision, read, and approved the submitted version.

## Conflict of Interest

The authors declare that the research was conducted in the absence of any commercial or financial relationships that could be construed as a potential conflict of interest.

## Publisher’s Note

All claims expressed in this article are solely those of the authors and do not necessarily represent those of their affiliated organizations, or those of the publisher, the editors and the reviewers. Any product that may be evaluated in this article, or claim that may be made by its manufacturer, is not guaranteed or endorsed by the publisher.
